# Siamese-Enhanced Multi-View Representation Learning for Robust Attack Detection in the Internet of Vehicles

**DOI:** 10.3390/s26196219

**Published:** 2026-09-30

**Authors:** Junhao Xie, Shuailing Yang, Xin Cai, Bo Wang, Bingfeng Xu

**Affiliations:** College of Information Science and Technology & Artificial Intelligence, Nanjing Forestry University, Nanjing 210037, China; junhaoxie@njfu.edu.cn (J.X.); yangsl@njfu.edu.cn (S.Y.); mswoo@njfu.edu.cn (X.C.); bowang@njfu.edu.cn (B.W.)

**Keywords:** Internet of Vehicles (IoV), multi-view learning, robustness, Siamese network, time-series attack detection

## Abstract

The rapid development of the Internet of Vehicles (IoV) has made cyberattacks targeting vehicular time-series data a critical security issue. Existing reconstruction-based detection methods mainly rely on global sequence modeling, which limits their effectiveness in highly dynamic vehicular environments and often results in missed detections of stealthy, localized attacks caused by subtle temporal variations. To address this limitation, we propose the Siamese-Enhanced Multi-View Representation Discrepancy (SMRD) framework for robust anomaly detection in vehicular networks. SMRD introduces a joint representation learning strategy that integrates global trend reconstruction with fine-grained local dynamic modeling. Specifically, the global view captures long-term temporal dependencies through sequence reconstruction and generates a reconstruction representation discrepancy. Meanwhile, the local view employs a novel intra-block and inter-block Siamese network to learn contextual relationships among adjacent time points and produce a Siamese representation discrepancy. Furthermore, to reduce representation bias caused by the non-stationary characteristics of IoV streaming data, an adaptive normalization strategy is introduced to stabilize input distributions during modeling and preserve essential statistical properties after reconstruction. By effectively combining the discrepancies from both views, SMRD enables comprehensive anomaly evaluation at each time point. Extensive experiments on the F2MD simulation platform and multiple real-world datasets, including SPMD, demonstrate that SMRD achieves state-of-the-art performance. Specifically, it achieves the highest F1-scores across all four simulated attack scenarios and delivers superior or competitive performance on public benchmarks, confirming its robustness for securing vehicular networks.

## 1. Introduction

As the Internet of Vehicles (IoV) rapidly expands, modern vehicular networks increasingly rely on continuous time-series data collected from various onboard sensors to support real-time perception, decision-making, and autonomous control [1]. These sensors continuously collect and transmit information about vehicle states, driving behavior, and surrounding road conditions [2]. However, the widespread exchange of such data through open Vehicle-to-Everything interfaces exposes vehicular systems to growing cybersecurity threats. Accordingly, misbehavior detection has become an essential security function in cooperative intelligent transportation systems. Haidar et al. experimentally assessed pseudonym-certificate management and a misbehavior-detection solution against Sybil and data-replay attacks in C-ITS [3]. More recently, Park et al. combined rule-based screening with deep-learning-based local misbehavior detection for Basic Safety Messages received by roadside units [4]. Malicious attackers can inject or manipulate sensor data, severely impairing a vehicle’s perception capability and decision-making. For example, researchers have injected falsified location data into Tesla Model S and Model 3 vehicles, leading to unsafe lane changes [5]. These incidents highlight the urgent need for effective methods to detect attacks on time-series data in dynamic vehicular environments.

In the field of Internet of Things (IoT) security, reconstruction-based generative models have become a widely adopted paradigm for time-series attack detection. These methods learn the underlying distribution of normal data and identify potential attacks by measuring the reconstruction discrepancy between the original input and the reconstructed output [6]. Early approaches, such as LSTM-Autoencoder [7], employed recurrent neural networks to capture temporal dependencies. However, their sequential processing nature limits the modeling of long-range dependencies and introduces considerable computational costs. To overcome these limitations, recent studies have increasingly adopted Transformer-based architectures. By leveraging self-attention mechanisms, Transformers can efficiently capture long-term dependencies with improved parallel computation efficiency. Furthermore, advanced frameworks have incorporated adversarial learning strategies to encourage anomalous samples to produce larger reconstruction errors, thereby improving the detection capability against complex spatiotemporal attacks [8].

Despite these advances, existing reconstruction-based methods still face a fundamental limitation. Most approaches model time-series data primarily from a global perspective. Although this strategy effectively captures overall trends, it often neglects fine-grained local dynamics and complex relationships among adjacent data points [9]. This limitation is particularly challenging in highly dynamic vehicular environments. In such environments, malicious attacks often appear as short-term and localized disturbances rather than significant global deviations [10]. Since conventional global models prioritize overall sequential consistency, they tend to smooth out these subtle anomalies.

**Figure 1:**
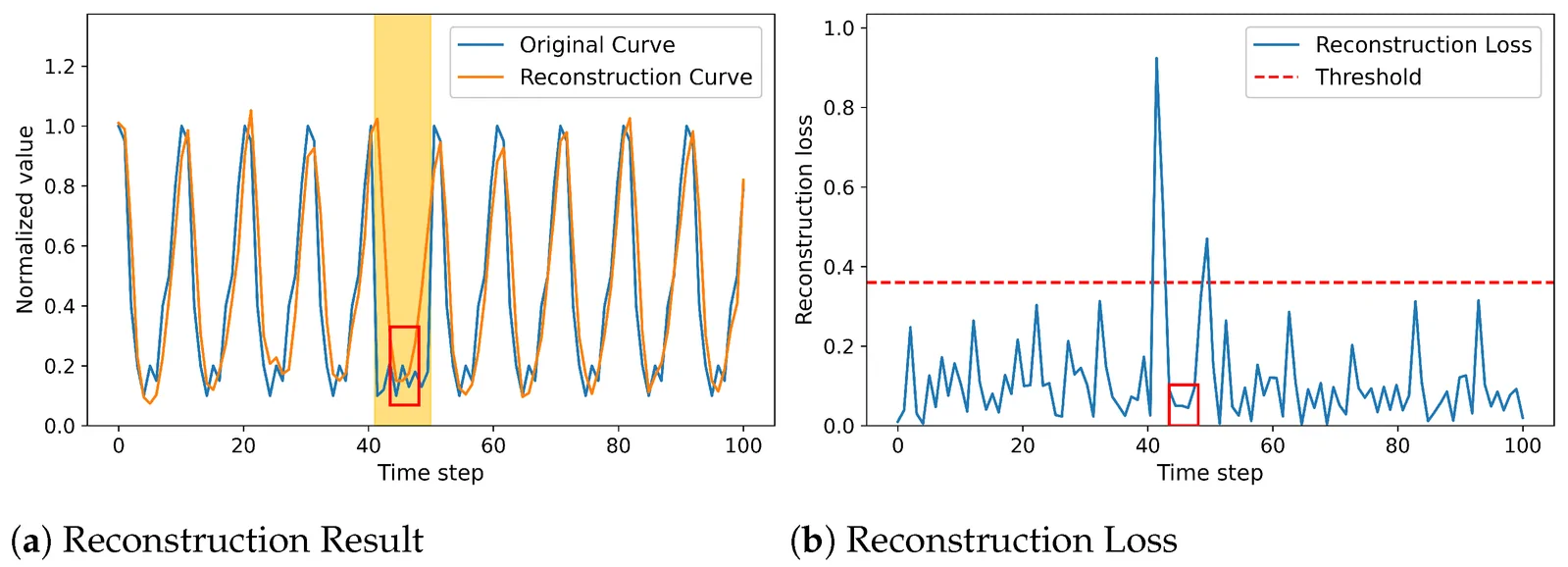
Failure case of a conventional reconstruction-based method in a highly dynamic environment. (**a**) Original and reconstructed sequences; (**b**) point-wise reconstruction loss. The yellow shaded region indicates the attack interval, the red box highlights the missed attack points, and the red dashed line denotes the detection threshold.

Figure 1 further demonstrates this limitation using a 100-step time-series example. The horizontal axis in both panels represents the time step, while the vertical axes represent the normalized sensor value and point-wise reconstruction loss in Figure 1a and Figure 1b, respectively. The yellow shaded region indicates the actual attack interval (approximately time steps 41–50), and the red box highlights the attack points missed by a conventional reconstruction-based method that relies only on global modeling. The abrupt changes at the two ends of the interval produce large reconstruction losses and are detected, whereas the small oscillations within the interval are similar in magnitude to normal troughs and are well reconstructed. Their reconstruction losses therefore remain below the detection threshold, and these attack points are classified as normal because their variations lie within the range of the globally learned normal pattern. This observation indicates that effective attack detection in highly dynamic IoV environments requires models that can simultaneously capture global trends and fine-grained local dependencies.

To address this challenge, this paper proposes the Siamese-Enhanced Multi-View Representation Discrepancy (SMRD) framework for robust time-series attack detection. The key motivation of our approach is that vehicular time series contain both global trends (long-term patterns) and local dynamics (short-term changes). By jointly modeling and integrating these two aspects, SMRD learns more comprehensive and robust feature representations. Specifically, SMRD introduces a dual-view representation learning strategy. From the global perspective, the framework reconstructs the original sequence to capture long-term temporal dependencies and generates a reconstruction representation discrepancy. Meanwhile, to overcome the limitations of global-only modeling, the local view introduces a novel intra-block and inter-block Siamese network. This dual-branch architecture measures representation similarity to capture fine-grained contextual variations among adjacent temporal points, thereby producing a Siamese representation discrepancy.

Furthermore, to reduce representation bias caused by the highly non-stationary characteristics of streaming vehicular data, we incorporate an adaptive normalization strategy into the framework. This mechanism first transforms the raw input into a more stable distribution for effective representation learning and then applies denormalization to the reconstructed output to recover important non-stationary characteristics. Finally, the discrepancies obtained from the global and local views are jointly integrated to generate a comprehensive anomaly score. Extensive experiments on the F2MD simulation platform and multiple real-world benchmarks demonstrate that SMRD achieves state-of-the-art performance. Moreover, comprehensive ablation studies verify the effectiveness of the proposed architecture and highlight the importance of the Siamese local view in detecting stealthy and localized attacks.

In summary, the main contributions of this paper are as follows:

1. We propose SMRD, a novel Siamese-Enhanced Multi-View Representation Discrepancy method for time-series attack detection in vehicular networks. By jointly optimizing a global reconstruction discrepancy and a local Siamese-based representation discrepancy, the proposed model effectively captures both long-term temporal dependencies and fine-grained local dynamics.
2. We design an adaptive normalization and denormalization strategy to reduce representation bias caused by non-stationary data. This strategy improves the model’s adaptability, detection capability, and robustness in highly dynamic vehicular environments.
3. We conduct extensive experiments on comprehensive benchmarks, including the F2MD simulation platform and multiple real-world datasets (e.g., SPMD, MSL). Experimental results demonstrate that SMRD consistently achieves the highest F1-scores across all four simulated attack scenarios and achieves leading performance on most public datasets. Detailed ablation studies further validate the effectiveness of each component, especially the Siamese local view.

The remainder of the paper is structured as follows: Section 2 reviews related studies on time-series anomaly detection. Section 3 introduces the architecture of the proposed SMRD framework. Section 4 presents the experimental setup and analyzes the comparative results. Finally, Section 5 concludes the paper and discusses future research directions.

## 2. Related Work

This section presents a structured review of existing studies, including general time-series anomaly detection methods and specialized attack detection approaches for the Internet of Vehicles (IoV). Furthermore, it discusses the limitations of current methods in jointly modeling global patterns and fine-grained local dynamics.

### 2.1. General Time-Series Anomaly Detection Methods

Early time-series anomaly detection methods mainly relied on traditional statistical and machine-learning techniques, including clustering, density estimation, and distance-based approaches [11,12,13]. Although computationally efficient, these methods are limited in modeling complex, high-dimensional, and nonlinear telemetry data. Deep generative and reconstruction-based models subsequently learned normal temporal representations from data. Representative methods, such as LSTM-VAE [14] and OmniAnomaly [15], detect anomalies by measuring reconstruction discrepancies. However, recurrent architectures incur sequential computation costs and have limited ability to capture long-range dependencies.

To overcome the limitations of recurrent architectures, attention mechanisms and Transformer-based models have been widely adopted for temporal representation learning. TranAD combines Transformer structures with adversarial training to enlarge reconstruction errors for anomalous samples [16]. The Anomaly Transformer detects abnormal temporal points through association discrepancies [17], while AnomalyBert uses pre-training to learn transferable temporal representations [18]. Beyond reconstruction-based approaches, CARLA employs contrastive learning for discriminative representations [19]. More recently, MAAT combines sparse association modeling with a selective Mamba state-space model to improve long-range dependency modeling and reduce computational redundancy [20].

Despite these advances, a fundamental modeling limitation remains: existing methods mainly optimize complete sequences, predefined association patterns, or single-view representations. As a result, short-duration and low-amplitude disturbances are often smoothed and hidden by dominant global patterns. Related observations have also been reported in communication-channel security. For example, in quantum key distribution systems, reliable synchronization-signal detection depends on probabilistic characteristics at the individual time-slot level [21], while interference with the calibration process may remain undetected when relying only on aggregate indicators such as the quantum bit error rate [22]. Such masking of local disturbances is particularly challenging for vehicular time series, where variables such as speed, acceleration, and position continuously change due to dynamic driving behaviors and road conditions. Therefore, effective attack detection in such environments requires models that can maintain global temporal consistency while explicitly capturing fine-grained relationships within local temporal regions.

### 2.2. Time-Series Attack Detection in the Internet of Vehicles

Time-series attack detection in IoV environments has evolved from rule-based filtering and state estimation toward data-driven temporal modeling. Early studies include CNN-KF, which combines convolutional features with Kalman filtering for real-time sensor anomaly detection [23], and DeepADV, which learns normal operational patterns in vehicular networks [24]. D-CNN-LSTM integrates convolutional and recurrent architectures to extract joint spatial–temporal features [25]. More recently, UltraADV targets lightweight unsupervised detection for V2X telemetry and edge deployment [26].

Recent IoV studies further explore multi-scale and structural temporal modeling. ET-DRF-GA uses an enhanced temporal convolutional network with genetic-algorithm optimization for connected automated vehicles [27], while MPGTrans uses a multi-path graph Transformer and semi-supervised learning for BSM misbehavior detection [28]. Although these methods improve detection through lightweight design, multi-scale features, or inter-vehicle structure, they mainly focus on global temporal patterns or relationships between vehicles.

To model diverse characteristics of sequential data, multi-view learning has recently attracted increasing attention [29]. Existing methods construct complementary views from temporal or graph-based representations [30]. However, vehicular time-series data are highly non-stationary, with dynamically changing distributions and spectral characteristics under different driving conditions. Methods relying on predefined view divisions may therefore struggle to distinguish normal variations from malicious local disturbances. Moreover, graph-based models capture relationships among vehicles or communication messages but lack explicit mechanisms for measuring fine-grained local consistency within an individual vehicle’s temporal sequence.

In summary, existing IoV attack detection methods mainly focus on global sequence modeling, computational efficiency, multi-scale feature extraction, or inter-vehicle topology analysis. They lack effective mechanisms for evaluating representation consistency among local temporal regions within individual vehicle sequences. The proposed SMRD framework addresses this limitation by integrating a global reconstruction view with a local Siamese view. The global view learns long-term temporal patterns, while the local view explicitly measures intra-block and inter-block representation consistency. By jointly combining global reconstruction discrepancies and local dynamic discrepancies, SMRD achieves more robust detection of stealthy and localized cyberattacks.

## 3. Model Introduction

In this section, we present the proposed Siamese-Enhanced Multi-View Representation Discrepancy (SMRD) framework. We first describe the overall architecture of the model. We then introduce the global reconstruction discrepancy, the local Siamese representation discrepancy, the adaptive normalization strategy, and the final loss function and anomaly score formulation.

### 3.1. Overall Architecture

**Figure 2:**
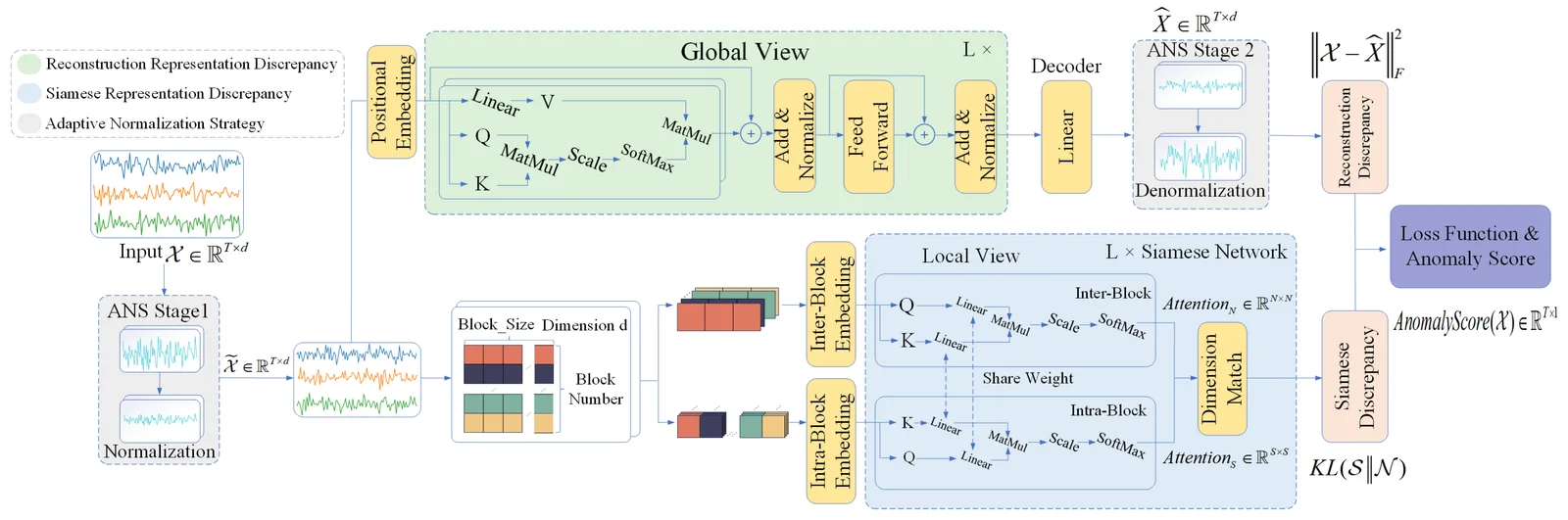
The overall architecture of the SMRD framework. Solid arrows denote the data flow, and dashed arrows denote weight sharing between the intra-block and inter-block branches. The background colors correspond to the three components shown in the legend.

The architecture of the SMRD model is illustrated in Figure 2. It consists of three main components: (i) Reconstruction Representation Discrepancy, which measures the reconstruction error between the original and reconstructed time series from a global perspective; (ii) Siamese Representation Discrepancy, which captures the discrepancy between the intra-block and inter-block branches from a local perspective; (iii) Adaptive Normalization Strategy, which dynamically normalizes and denormalizes the input data to reduce representation bias caused by non-stationary information.

The input sequence $X\in R^{T\times d}$ is a multivariate time series with *T* time steps and *d* variables. The SMRD framework first applies adaptive normalization to reduce the influence of non-stationary components. The normalization sequence is then processed from both global and local perspectives. In the global view, the model reconstructs the time series to capture long-term dependencies. The reconstructed sequence is subsequently denormalized, and the reconstruction representation discrepancy is calculated. In the local view, the sequence is divided into sub-blocks $X\in R^{S\times N\times d}$. An intra-block and inter-block Siamese network is employed to model local dynamic dependencies and compute the Siamese representation discrepancy. During training, these two discrepancies jointly contribute to the optimization objective. During inference, they are combined to generate anomaly scores. Time segments with scores exceeding the threshold, which is set to the (100−δ)-th percentile of the combined anomaly scores of the training and test sets with a preset percentage δ, are identified as potential attacks.

### 3.2. Reconstruction Representation Discrepancy

Reconstruction-based methods assume that normal and anomalous data exhibit distinct characteristics. Consequently, a model trained on normal data can reconstruct normal patterns accurately but struggles to reconstruct anomalous patterns. By comparing the reconstructed sequence with the original input, time points with large reconstruction errors can be identified as potential attacks. The SMRD framework employs a self-attention mechanism to reconstruct the input time series. The architecture alternates between attention layers and feed-forward layers. This design enables the model to capture global dependencies and assign greater attention to regions that are important for reconstruction [31].

Assuming the input multivariate time series is $X\in R^{T\times d}$, the encoding process at the *l*-th level is defined as:

(1) $$\begin{array}{cc} \; & Z^{l}=\mathrm{Layer}-\mathrm{Norm}\left(\mathrm{Attention}\left(X^{l-1}\right)+X^{l-1}\right)\\ \; & X^{l}=\mathrm{Layer}-\mathrm{Norm}\left(\mathrm{Feed}-\mathrm{Forward}\left(Z^{l}\right)+Z^{l}\right)\\ \; & X^{0}=\mathrm{Positional}-\mathrm{Embedding}\left(X\right)\\ \; & {\hat{X}}^{*}=\mathrm{Linear}\left(Z^{l}\right) \end{array}$$

where $Z^{l}\in R^{T\times d_{model}},1\leq l\leq L$ denotes the hidden layer representation result at the *l*-th layer, $X^{l}\in R^{T\times d_{model}},1\leq l\leq L$ represents the output at the *l*-th layer, and ${\hat{X}}^{*}\in R^{T\times d}$ represents the time series after global view reconstruction. The self-attention mechanism Attention(·) in the *l*-th layer is formulated as:

(2) $$\begin{array}{cc} \; & \mathrm{Init}:\;Q,K,V=X^{l-1}W_{Q}^{l},X^{l-1}W_{K}^{l},X^{l-1}W_{V}^{l}\\ \; & \mathrm{Association}:\;S^{l}=\mathrm{Softmax}\left(\frac{QK^{T}}{\sqrt{d_{model}}}\right)\\ \; & \mathrm{Reconstruction}:\;{\hat{Z}}^{l}=S^{l}V \end{array}$$

where $Q,K,V\in R^{T\times d_{model}}$ denote the query, key, and value in the self-attention mechanism; $W_{Q}^{l}$, $W_{K}^{l}$, and $W_{V}^{l}$ are the learnable projection matrices in the *l*-th layer; and ${\hat{Z}}^{l}\in R^{T\times d_{model}}$ is the hidden representation produced by that layer. For multi-head attention, Q, K, and V are divided into $Q_{m},K_{m},V_{m}\in R^{T\times \frac{d_{model}}{H}}$, and the corresponding output is ${\hat{Z}}_{m}^{l}\in R^{T\times \frac{d_{model}}{H}}$.

### 3.3. Siamese Representation Discrepancy

Reconstruction-based methods primarily focus on reconstructing time-series data from a global perspective through the encoder–decoder architecture. Although effective in capturing overall patterns, such approaches often have limited ability to model the fine-grained local dynamics embedded in time-series data. Our observations indicate that normal data points usually share common underlying patterns and exhibit strong local correlations. As a result, when viewed from different local perspectives, normal points tend to present similar feature representations. Therefore, the representation discrepancy among different local perspectives is generally small, reflecting high representation consistency. In contrast, anomalous points are rare and often lack stable underlying patterns. Consequently, they exhibit weak similarity to neighboring points, leading to larger representation discrepancies across local perspectives and lower representation consistency.

Motivated by this observation, we design an intra-block and inter-block Siamese network to capture fine-grained local correlation patterns in time-series data. Before introducing Siamese Representation Discrepancy, we define two key concepts: intra-block and inter-block. The intra-block consists of time nodes located at the same temporal position across different blocks, capturing local variations at corresponding positions. In contrast, the inter-block contains all time nodes within a block, enabling the modeling of internal temporal dynamics within local segments. Unlike the global view, which focuses on overall sequence behavior, the local view explores detailed local information. By constructing a pair of Siamese branches for intra-block and inter-block representations, we aim to characterize the representation discrepancy between different local structures. After partitioning the time series into blocks, the intra-block branch groups time nodes from different blocks that share the same position, while the inter-block branch groups all time nodes within each block. Correlation patterns are then learned within each branch. The Siamese representation discrepancy is obtained by comparing the similarity between the learned representations of the two branches. This design enables the model to distinguish normal and anomalous points by exploiting local dynamic information.

**Figure 3:**
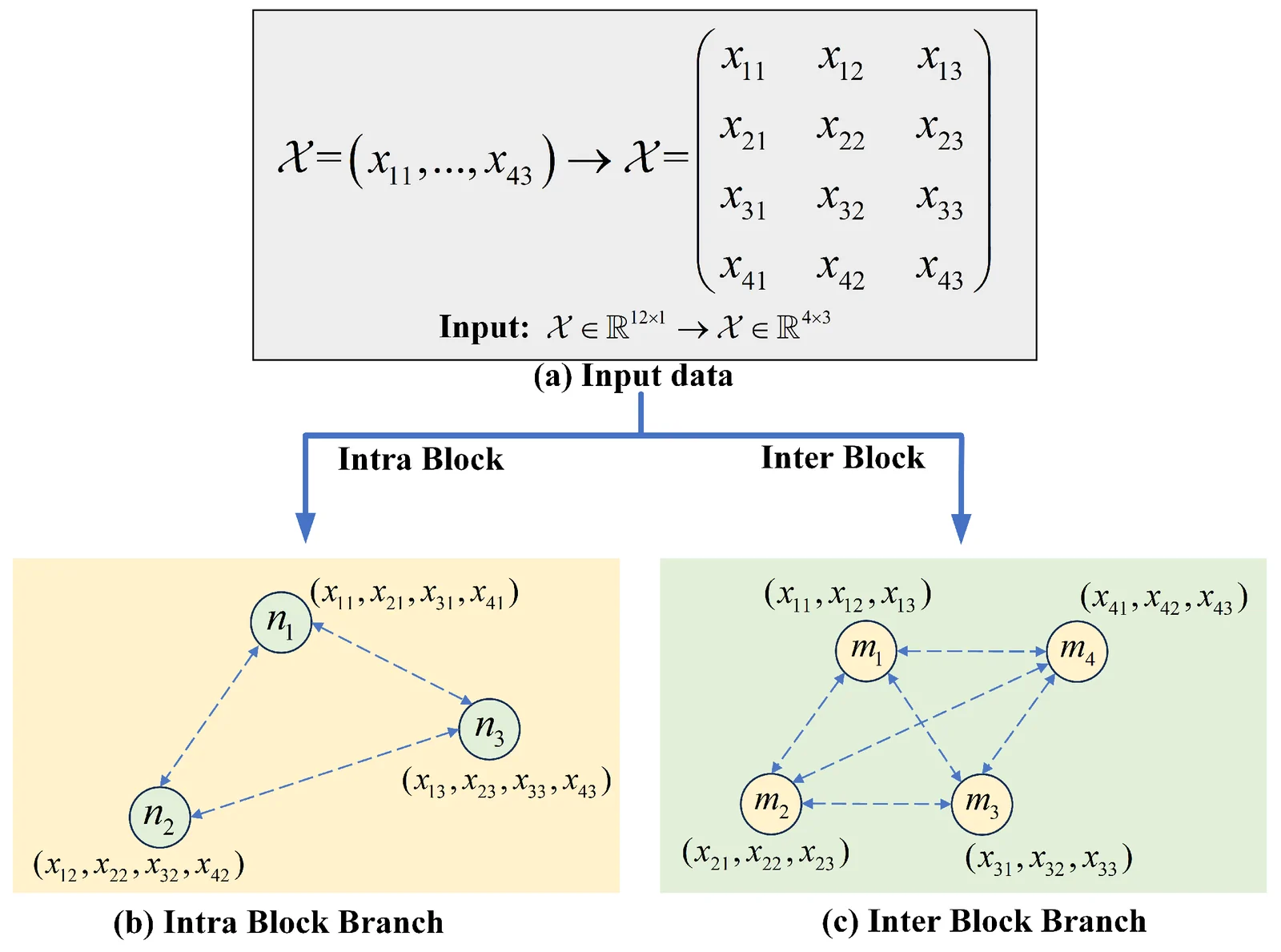
An example of intra-block branch and inter-block branch structures. (**a**) Input data $X\in R^{12\times 1}\to X\in R^{4\times 3}$. (**b**) Intra-block branch, where each group consists of time nodes from different blocks at the same position. Dashed lines denote correlation patterns between groups. (**c**) Inter-block branch, where each group contains all time nodes within a block. Dashed lines denote correlation patterns between groups.

The procedures of the intra-block and inter-block branches are illustrated in Figure 3. The figure presents a simple example for a one-dimensional time series. We further extend this process to a *d*-dimensional sequence and summarize the corresponding partitioning procedure in Algorithm 1. The main idea is to transform the time series into a row-major tensor through a reshaping operation. After positional embedding, the reshaped tensor is decomposed into two complementary structures that capture intra-block and inter-block relationships, respectively. This design provides flexible and informative inputs for the subsequent Siamese network.
**Algorithm 1:** Time series block partitioning.
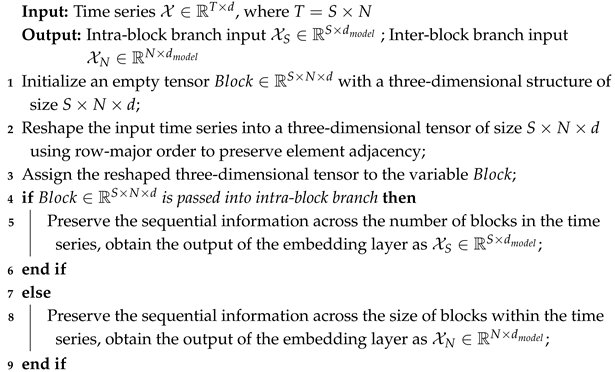



The intra-block and inter-block Siamese network contains multiple encoding layers. For simplicity, we describe one encoding layer as an example. After block partitioning, positional embedding is applied to both branches. In the intra-block branch, the positional embedding preserves the sequential information across blocks, resulting in the representation $X_{S}\in R^{S\times d_{model}}$. In the inter-block branch, the positional embedding preserves the sequential information within each block, resulting in $X_{N}\in R^{N\times d_{model}}$. The representations learned by the two branches are defined as follows:

(3) $$\begin{array}{cc} \; & Q_{S},K_{S},V_{S}=X_{S}^{l-1}W_{Q}^{l},X_{S}^{l-1}W_{K}^{l},X_{S}^{l-1}W_{V}^{l},1\leq l\leq L\\ \; & Q_{N},K_{N},V_{N}=X_{N}^{l-1}W_{Q}^{l},X_{N}^{l-1}W_{K}^{l},X_{N}^{l-1}W_{V}^{l},1\leq l\leq L\\ \; & \mathrm{Intra}-\mathrm{Block}:\;{\mathrm{Attention}}_{S}=\mathrm{Softmax}\left(\frac{Q_{S}K_{S}^{T}}{\sqrt{d_{\mathrm{model}}}}\right)\\ \; & \mathrm{Inter}-\mathrm{Block}:\;{\mathrm{Attention}}_{N}=\mathrm{Softmax}\left(\frac{Q_{N}K_{N}^{T}}{\sqrt{d_{\mathrm{model}}}}\right) \end{array}$$

where $Q_{S},K_{S},V_{S}\in R^{S\times d_{model}}$ and $Q_{N},K_{N},V_{N}\in R^{N\times d_{model}}$ denote the query, key, and value matrices in the intra-block and inter-block branches, respectively. ${\mathrm{Attention}}_{S}\in R^{S\times S}$ and ${\mathrm{Attention}}_{N}\in R^{N\times N}$ represent the corresponding attention matrices. The two branches share the same projection matrices, including the query and key projections $W_{Q}^{l}$ and $W_{K}^{l}$, which map their inputs into a common projection space. This weight-sharing mechanism is a key characteristic of a Siamese network. It enables both branches to learn representations within a common feature space, improving their ability to capture structural similarities and discrepancies. Such a design enhances both representation capability and generalization performance.

Although the proposed Siamese network effectively captures local dynamics, several challenges remain. The intra-block branch ignores dependencies across different blocks, while the inter-block branch does not explicitly model relationships among corresponding positions in different blocks. Moreover, the outputs of the two branches have different dimensions, making direct comparison difficult. Therefore, the representations must be aligned before calculating the Siamese representation discrepancy. To address this issue, we apply bilinear interpolation to the attention matrices generated under different block sizes and then average the interpolation results across multiple scales. Compared with conventional multi-scale processing [32], this strategy effectively exploits information from multiple block configurations. It preserves fine-grained details and local structures while producing smooth transitions. In addition, bilinear interpolation provides a favorable balance between performance and computational efficiency. Assuming that the values of Attention(·) are known at four neighboring points $Q_{11}\;=\;\left(i,j\right)$, $Q_{12}\;=\;\left(i,j+1\right)$, $Q_{21}\;=\;\left(i+1,j\right)$ and $Q_{22}\;=\;\left(i+1,j+1\right)$, the bilinear interpolation process is defined as:

(4) $$\begin{array}{cc} \; & \mathrm{Attention}\left(i+u,j+v\right)=\mathrm{Interpolate}\left(Q_{11},Q_{12},Q_{21},Q_{22}\right)\\ \; & =\left(1-u\right)\left(1-v\right)\mathrm{Attention}\left(Q_{11}\right)+u\left(1-v\right)\mathrm{Attention}\left(Q_{21}\right)\\ \; & \;+\left(1-u\right)v\mathrm{Attention}\left(Q_{12}\right)+uv\mathrm{Attention}\left(Q_{22}\right) \end{array}$$

where Interpolate(·) denotes bilinear interpolation, while *u* and *v* represent offsets along the horizontal and vertical directions, respectively.

After averaging information across multiple scales, the final representations are obtained as follows:

(5) $$\begin{array}{cc} S & =\frac{1}{M}\sum_{m=1}^{M}\mathrm{Interpolate}({\mathrm{Attention}}_{S}^{c(m)}) \end{array}$$

(6) $$\begin{array}{cc} N & =\frac{1}{M}\sum_{m=1}^{M}\mathrm{Interpolate}({\mathrm{Attention}}_{N}^{c(m)}) \end{array}$$

where *M* denotes the number of block sizes, and ${\mathrm{Attention}}_{S}^{\left(m\right)}$ and ${\mathrm{Attention}}_{N}^{\left(m\right)}$ denote the attention matrices obtained with the *m*-th block size. The interpolation operation maps each attention matrix to a common size of T×T according to the temporal positions of the original window. Consequently, $S\in R^{T\times T}$ and $N\in R^{T\times T}$ represent the aligned and multi-scale aggregated representations of the intra-block and inter-block branches, respectively. After alignment, the *t*-th row of each matrix describes the associations between time point *t* and all time points in the window from the intra-block and inter-block perspectives, respectively. Therefore, Equation (7) compares the aligned representations rather than the raw branch outputs, enabling the Siamese network to measure structural discrepancies between the two complementary temporal views.

To construct a robust discrepancy measure, we aggregate the representations from multiple encoding layers. Specifically, the Siamese representation discrepancy is computed by averaging the discrepancies across all hierarchical layers. This design enables the model to leverage feature information at different semantic levels and obtain a more comprehensive representation. The Siamese representation discrepancy is defined as:

(7) $$\mathrm{SRDis}\left(S,N;X\right)=\frac{1}{L}\sum_{l=1}^{L}\left[KL\left(S^{l}∥\mathrm{Stopgrad}(N^{l})\right)+KL(N^{l}∥\mathrm{Stopgrad}(S^{l}))\right]\;$$

where SRDis(S,N;X) denotes the Siamese representation discrepancy and KL(·∥·) represents the Kullback–Leibler divergence. This divergence measures the difference between the distributions of S and N. To improve training stability, the stop-gradient operation is applied to both branches [33]. This strategy decouples gradient propagation between the intra-block and inter-block branches during backpropagation, allowing them to update independently and reducing gradient interference.

**Figure 4:**
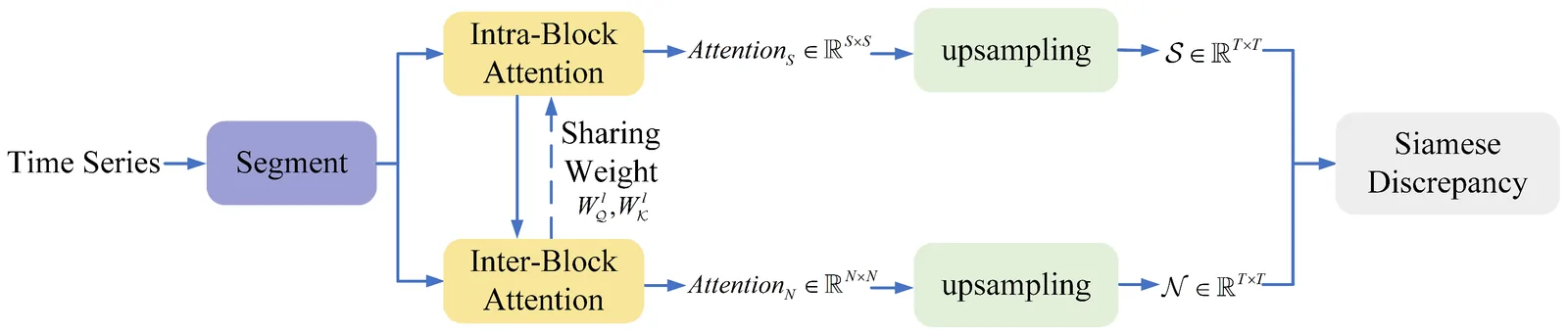
The overall process of Siamese network branches. Solid arrows denote the data flow, and the arrows between the two attention modules indicate that they share the projection weights $W_{Q}^{l}$ and $W_{K}^{l}$.

Based on the above design, Figure 4 presents the overall workflow of the intra-block and inter-block Siamese network. The input time series is first partitioned into intra-block and inter-block branches through the blocking operation shown in Figure 3. The attention mechanism is then applied to both branches using shared parameters $W_{Q}^{l}$ and $W_{K}^{l}$, producing the attention matrices ${\mathrm{Attention}}_{S}\in R^{S\times S}$ and ${\mathrm{Attention}}_{N}\in R^{N\times N}$. These matrices are subsequently upsampled to the same resolution, resulting in $S\in R^{T\times T}$ and $N\in R^{T\times T}$. Finally, the discrepancies between the two branches are quantified through multi-layer KL divergence, forming the Siamese representation discrepancy.

### 3.4. Adaptive Normalization Strategy

**Figure 5:**
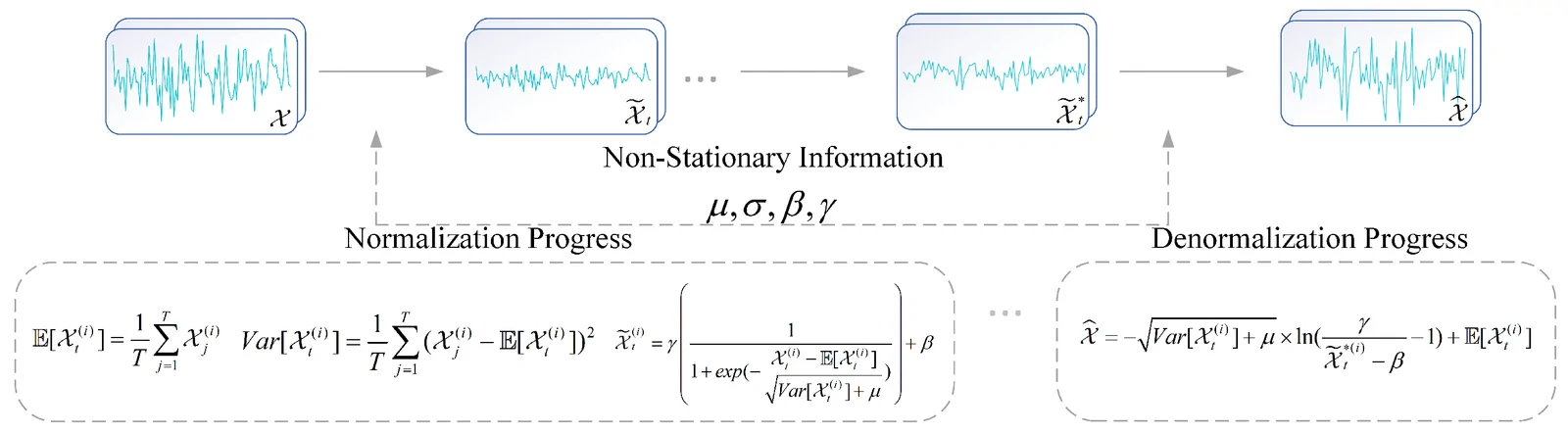
Adaptive normalization strategy framework. Solid arrows denote the data flow, and dashed arrows indicate that the non-stationary statistics (μ, σ, β, γ) obtained during normalization are reused during denormalization. The ellipses denote the intermediate reconstruction steps, which are omitted for brevity.

Due to the highly dynamic nature of the IoV and the variability in vehicle behavior, vehicular time-series data often exhibit significant fluctuations and strong non-stationary characteristics. As a result, their statistical properties, such as means and variances, may change over time. These non-stationary characteristics can affect the learning of correlation patterns and introduce representation bias, which may reduce the accuracy of the learned representations [34]. To address this issue, we propose an adaptive normalization strategy consisting of two stages: normalization and denormalization. The normalization stage transforms the original time series into a stationary distribution by removing non-stationary information, such as variations in the mean and variance. To preserve important non-stationary characteristics, the denormalization stage restores the removed statistical information to the reconstructed sequence. Since non-stationary information does not affect the calculation of the Siamese representation discrepancy, normalization is applied to both the global and local views, whereas denormalization is applied only to the global view. The overall framework is illustrated in Figure 5.

During the normalization stage, an adaptive normalization strategy is applied to the input time series $X\in R^{T\times d}$ to remove non-stationary information, such as mean and variance. This process ensures that the normalized sequence maintains more consistent statistical properties across different time intervals. The mean and variance of the time series are calculated as follows:

(8) $$\begin{array}{cc} E[X_{t}^{\left(i\right)}] & =\frac{1}{T}\sum_{j=1}^{T}X_{j}^{\left(i\right)}\\ Var[X_{t}^{\left(i\right)}] & =\frac{1}{T}\sum_{j=1}^{T}{\left(X_{j}^{\left(i\right)}-E\left[X_{t}^{\left(i\right)}\right]\right)}^{2} \end{array}$$

where $X_{j}^{\left(i\right)}$ denotes the value of the *j*-th time point (0≤j≤T) in the *i*-th (0≤i≤d) dimension, and $E[X_{t}^{\left(i\right)}]$ and $Var[X_{t}^{\left(i\right)}]$ denote the mean and variance, respectively.

Using these statistical parameters, the normalization process is defined as follows:

(9) $${\tilde{X}}_{t}^{\left(i\right)}=\gamma \left(\frac{1}{1+\exp(-\frac{X_{t}^{\left(i\right)}-E\left[X_{t}^{\left(i\right)}\right]}{\sqrt{Var[X_{t}^{\left(i\right)}]+\mu }})}\right)+\beta$$

where γ and β are learnable affine parameters, μ is a small perturbation term for numerical stability, and ${\tilde{X}}_{t}^{\left(i\right)}$ denotes the normalized time series. After normalization, the sequence exhibits more stable statistical properties. The removal of non-stationary information makes the data more stationary, meaning that the average level and variance remain relatively consistent throughout the sequence.

During reconstruction, the SMRD model takes the normalized sequence ${\tilde{X}}_{t}^{\left(i\right)}$ as input and generates the reconstructed sequence ${\tilde{X}}_{t}^{*(i)}$. To preserve the non-stationary characteristic of the original data, we design a denormalization stage that is symmetric to the normalization process. This stage restores the removed statistical information to the reconstructed sequence. Specifically, the denormalization process is defined as follows:

(10) $$\hat{X}=-\sqrt{Var[X_{t}^{\left(i\right)}]+\mu }\times \ln\left(\frac{\gamma }{{\tilde{X}}_{t}^{*(i)}-\beta }-1\right)+E\left[X_{t}^{\left(i\right)}\right]$$

where the same statistical parameters used in Equation (9) are employed. $\hat{X}$ denotes the final denormalized reconstruction result. Combined with the reconstruction mechanism introduced in Section 3.2, the reconstruction representation discrepancy is defined as:

(11) $$\mathrm{RRDis}\left(X\right)={∥X-\hat{X}∥}_{F}^{2}$$

where RRDis(X) denotes the reconstruction representation discrepancy, ${∥\cdot ∥}_{F}$ denotes the Frobenius norm, and X and $\hat{X}$ represent the original input time series and the denormalized reconstructed time series, respectively.

### 3.5. Loss Function and Anomaly Score Definition

Based on the global view and local view, the proposed SMRD framework generates two complementary discrepancy measures: the reconstruction representation discrepancy and the Siamese representation discrepancy. These two discrepancies are jointly utilized to optimize the model. The reconstruction representation discrepancy encourages the model to capture informative global correlations within the time series, whereas the Siamese representation discrepancy guides the model to focus on fine-grained local dynamics. By integrating these complementary objectives, the model can effectively learn both global and local correlation patterns, resulting in more informative and discriminative representations [17]. The overall loss function of the SMRD model is defined as follows:

(12) $$L_{Total}\left(S,N,\lambda ;X\right)=\mathrm{RRDis}\left(X\right)+\lambda \times {∥\mathrm{SRDis}(S,N;X)∥}_{1}$$

where RRDis(X) denotes the reconstruction representation discrepancy, SRDis(S,N;X) denotes the Siamese representation discrepancy, and ${∥\cdot ∥}_{1}$ represents the L1 norm. The parameter λ>0 controls the contribution of the Siamese representation discrepancy to the overall loss. The optimal value of λ is determined through a randomized search strategy, which automatically explores the parameter space to achieve an appropriate balance between the reconstruction and Siamese discrepancy.

To further exploit the complementary information provided by the two views, we jointly use the normalized Siamese representation discrepancy and the reconstruction representation discrepancy to define the final anomaly score. The anomaly score is calculated as follows:

(13) $$\mathrm{AnomalyScore}\left(X\right)=\mathrm{Softmax}\left(\mathrm{SRDis}(S,N;X)\right)⊙\mathrm{RRDis}\left(X\right)$$

where ⊙ denotes element-wise multiplication, and $\mathrm{AnomalyScore}\left(X\right)\in R^{T\times 1}$ represents the anomaly score produced by SMRD for the time series $X\in R^{T\times d}$. In general, anomalous points exhibit larger reconstruction and Siamese representation discrepancies than normal points. Element-wise multiplication therefore amplifies anomaly-related signals while suppressing normal patterns, strengthening the interaction between the global and local views.

## 4. Experiment

This section presents a comprehensive empirical evaluation of the proposed SMRD framework. We first introduce the simulation environment, attack configurations, and dataset construction process. Then, we describe the baseline methods and evaluation metrics used for comparison. Finally, we provide implementation details and analyze the experimental results under various vehicular cyberattack scenarios.

### 4.1. Simulation Tool and Datasets

Due to the limitations of the widely adopted VeReMi benchmark [35], which does not provide sufficient time-series data for different vehicle models, we employed the Framework for Misbehavior Detection (F2MD) [36] to generate data for evaluating the proposed method. F2MD provides a dynamic V2X simulation environment that supports various misbehavior attacks and enables real-time temporal data collection. In our experimental setup, we continuously collect and aggregate Basic Safety Messages (BSMs), which represent the primary information exchange medium in IoV networks. These messages contain spatiotemporal information, including vehicle position, velocity, and acceleration, from both benign and malicious vehicles. The simulations use the LuST scenario, which is based on the real road network and traffic demand of Luxembourg City, and vehicle communication is implemented using IEEE 802.11p. The detailed simulation configurations of F2MD are summarized in Table 1.

To evaluate the robustness of SMRD against both stealthy and aggressive attacks, we construct four representative cyberattack scenarios in F2MD: Constant Position Offset (CPO), Disruptive DoS with Sybil spoofing (DDS), Data Replay with Sybil spoofing (DRS), and Random DoS with Sybil spoofing (DRDS). These attacks introduce different types of temporal and structural perturbations into vehicular telemetry streams, as described in Table 2. Specifically, the four attacks represent persistent value offset, abnormal message frequency with identity switching, temporal replay, and data randomization, respectively.

### 4.2. Baselines and Evaluation Criteria

To comprehensively evaluate the effectiveness of SMRD, we compare it with 12 representative anomaly detection methods. These methods include the classical approaches iForest [37] and OCSVM [38]; the reconstruction-based models OmniAnomaly [15], MAD-GAN [39], CAE-M [40], and TranAD [16]; the multi-view models AnomalyTrans [17] and DTAAD [41]; the density-based model DAGMM [42]; the pre-training-based model AnomalyBert [18]; and the IoV-oriented methods UltraADV [26] and ET-DRF-GA [27]. To ensure a fair comparison, all baselines are configured according to the recommendations in their original papers or official implementations and are evaluated using the same data splits, window size, and evaluation protocol as SMRD.

Beyond conventional point-based evaluation metrics, including Precision, Recall, and F1-score, we adopt additional composite metrics to comprehensively evaluate temporal detection performance. These metrics include [6] the following:

1. The affiliation precision/recall pair, which evaluates the consistency between predicted anomalies and ground-truth anomaly regions.
2. Range-AUC-ROC and Range-AUC-PR, which assess detection performance under range-based label transformations using ROC and PR curves, respectively.
3. V_ROC and V_PR, which measure the volume under the ROC and PR curves, respectively, providing a more comprehensive evaluation of detection performance.

### 4.3. Implementation Details

The backbone architecture of SMRD is configured with L=3 encoder layers, a hidden representation dimension of 256, and H=2 parallel attention heads. The multi-task loss balancing coefficient λ is set to 1. Model parameters are optimized using the Adam optimizer with an initial learning rate of $10^{-4}$ and default momentum settings. The model is trained with a batch size of 64 for a maximum of 5 epochs. These settings are fixed across all experiments on the F2MD scenarios and public benchmarks.

For each F2MD scenario, the training set contains only normal vehicle messages, whereas attack messages are introduced exclusively in the test set. The anomaly threshold is determined as the (100−δ)-th percentile of the combined anomaly scores from the training and test sets, where δ denotes the threshold percentage. All four attack scenarios use the same Block_Size =[3,5] and Win_Size =60. The threshold percentage δ is set to 2% for DRDS and 1% for the other three scenarios. The dataset-specific settings are summarized in Table 3.

To evaluate temporal detection capability of SMRD under realistic attack scenarios, we adopt the standard Point-Adjustment (PA) protocol [17,18]. Under PA, if any point within a consecutive anomalous segment is detected, the entire segment is labeled as an attack. This protocol evaluates whether an anomaly segment can be identified, rather than requiring every anomalous point to be correctly localized.

### 4.4. Main Results

#### 4.4.1. Comparative Experiments on Vehicular Attack Scenarios

To evaluate the effectiveness of the proposed SMRD framework, we first conduct comparative experiments under four multivariate time-series vehicular attack scenarios: CPO, DDS, DRS, and DRDS. The performance is compared with 12 representative baseline models using Precision, Recall, and F1-score. The quantitative results are summarized in Table 4. SMRD achieves superior overall performance, obtaining F1-scores of 98.79%, 97.59%, 98.40%, and 98.35% on CPO, DDS, DRS, and DRDS, respectively. Compared with the strongest baseline method in each scenario, SMRD improves the F1-score by 0.37, 0.27, 0.29, and 5.20 percentage points, respectively. In particular, SMRD significantly outperforms traditional methods iForest, OCSVM, and DAGMM. These improvements demonstrate the effectiveness of jointly modeling global structural and local temporal dynamics for vehicular anomaly attack detection.

To provide a more comprehensive evaluation, we further compare SMRD with three strong baseline models, namely AnomalyBert, AnomalyTrans, and DTAAD, using additional evaluation metrics. Table 5 is obtained from the same experimental runs as Table 4, with identical models, data splits, and experimental settings; only the evaluation metrics differ. As reported in Table 5, SMRD outperforms AnomalyBert, AnomalyTrans, and DTAAD on most evaluation metrics. This advantage mainly stems from SMRD’s ability to capture fine-grained temporal dependencies among adjacent timestamps, together with the adaptive normalization strategy that effectively reduces representation bias caused by non-stationary noise. Although AnomalyBert achieves higher Aff-P and Aff-R in several scenarios, this advantage is mainly attributed to its explicit synthetic anomaly injection during training, which directly optimizes boundary-related evaluation metrics. In contrast, SMRD relies on fully unsupervised multi-view representation learning and provides a better balance among point-based, range-based, and volume-based detection performance, demonstrating stronger robustness and generalization ability.

#### 4.4.2. Generalization Across Public Real-World Benchmarks

To further evaluate the cross-domain generalization capability of the proposed framework beyond specialized vehicular attack scenarios, we conducted extensive comparative experiments on three publicly available IoT and space telemetry benchmarks:

1. **Security Pilot Model Deployment (SPMD):** A real-world traffic dataset containing vehicle dynamics and sensor measurements. Following [43], we extract vehicle speed, GPS speed, and acceleration signals sampled at 100 ms intervals to simulate realistic Internet of Vehicles (IoV) physical scenarios, and inject anomalies into these signals.
2. **Mars Science Laboratory (MSL) [44]:** A multivariate telemetry dataset collected from NASA Mars rovers, consisting of complex and continuous sensor measurements.
3. **Soil Moisture Active Passive (SMAP) [45]:** A dataset containing soil observation data and aerospace telemetry signals. Compared with MSL, SMAP contains a larger proportion of abrupt point anomalies.

The performance of SMRD is compared with ten representative time-series anomaly detection methods widely used in IoT and industrial scenarios, including Adformer [46], MUTANT [47], TranAD [16], AnomalyTrans [17], THOC [48], InterFusion [49], OmniAnomaly [15], BeatGAN [50], CARLA [19], and MAAT [20]. The detailed comparison results are reported in Table 6.

As shown in Table 6, SMRD achieves state-of-the-art performance on both SPMD and SMAP, obtaining F1-scores of 94.25% and 98.94%, respectively. On the MSL telemetry benchmark, SMRD achieves the second-best performance with an F1-score of 94.68%, closely approaching MAAT (95.09%) with only a 0.41-percentage-point gap. These results demonstrate the strong transferability of SMRD across diverse physical domains, ranging from intelligent transportation systems to aerospace telemetry, without requiring domain-specific parameter adjustment.

#### 4.4.3. Robustness Under High Attack Density

To evaluate the robustness of SMRD under severe attack conditions, we increase the attack density in the test sets to 40%, 50%, and 60% for all four attack types. The training set contains only normal data, while the model parameters and threshold percentage δ remain unchanged throughout the experiment. Thus, the results reflect the effect of increasing attack density on a fixed model.

**Figure 6:**
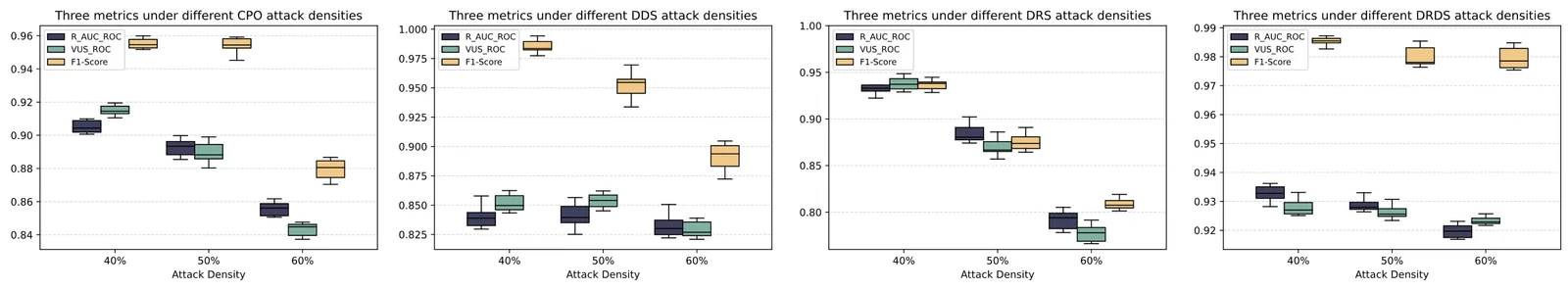
Model’s robustness to high-density attacks.

As shown in Figure 6, the average F1-score across the four attack types decreases from 96.52% at 40% attack density to 88.97% at 60%. However, the degradation varies considerably across attack types. The F1-score for DRDS remains nearly unchanged, decreasing from 98.51% to 97.95%, whereas DRS shows the largest degradation, from 93.67% to 80.88%. The F1-scores for CPO and DDS decrease from 95.54% and 98.38% to 87.94% and 89.11%, respectively.

This difference is mainly related to the local-context assumption underlying the Siamese representation discrepancy. DRS replays previously observed messages whose values generally remain within the normal range and is therefore detected mainly through local temporal inconsistency. As replayed messages occupy a larger portion of a window, the available normal context decreases, weakening the discrepancy between the intra-block and inter-block representations. In contrast, DRDS generates randomized values that remain substantially different from normal behavior, allowing the local discrepancy to remain pronounced even at high attack densities. For CPO, the performance degradation at high density is partly due to adaptive normalization, which can absorb a persistent position offset when the attack occupies most of a window.

Overall, increasing attack density generally reduces detection performance, but the magnitude of degradation depends on the temporal characteristics of the attack and its effect on the local context. When attacks dominate a temporal window, the assumption that anomalous behavior constitutes a local deviation from normal behavior becomes less valid. Nevertheless, the F1-score remains above 80% for all four attack types even at a density of 60%, indicating that SMRD retains substantial detection capability under severe attack conditions.

### 4.5. Ablation Study

To systematically investigate the contributions of the Reconstruction Representation Discrepancy (RRDis), Siamese Representation Discrepancy (SRDis), and Adaptive Normalization strategy, we perform ablation experiments on seven structural variants of SMRD. The detection performance is evaluated using the F1-score, with the results summarized in Table 7.

As shown in Table 7, the complete SMRD framework consistently outperforms all reduced variants across the four attack scenarios, achieving the highest average F1-score of 98.28%. The main observations are summarized as follows:

1. **Effectiveness of Adaptive Normalization:** Introducing bilateral adaptive normalization provides considerable performance improvements. Specifically, it increases the average F1-score from 67.29% to 71.48% in the reconstruction-only configuration and from 92.73% to 98.28% in the complete dual-branch architecture. This demonstrates its ability to reduce non-stationary variations and improve feature distribution alignment in vehicular time series.
2. **Complementarity of Multi-View Discrepancies:** Incorporating the Siamese representation branch into the normalized reconstruction framework improves the average F1-score by 26.80 percentage points (from 71.48% to 98.28%). This significant improvement verifies that the local temporal patterns captured by SRDis and the global reconstruction information modeled by RRDis provide complementary representations for anomaly detection.
3. **Effect of the Siamese form:** Replacing the Siamese branch with a standard attention branch reduces the average F1-score from 98.28% to 92.63%, and the F1-score decreases on all four attack types. This indicates that the gain of SMRD cannot be attributed merely to an additional attention branch; the proposed intra-/inter-block Siamese form plays a key role.

### 4.6. Model Analysis

#### 4.6.1. Parameter Sensitivity

**Figure 7:**
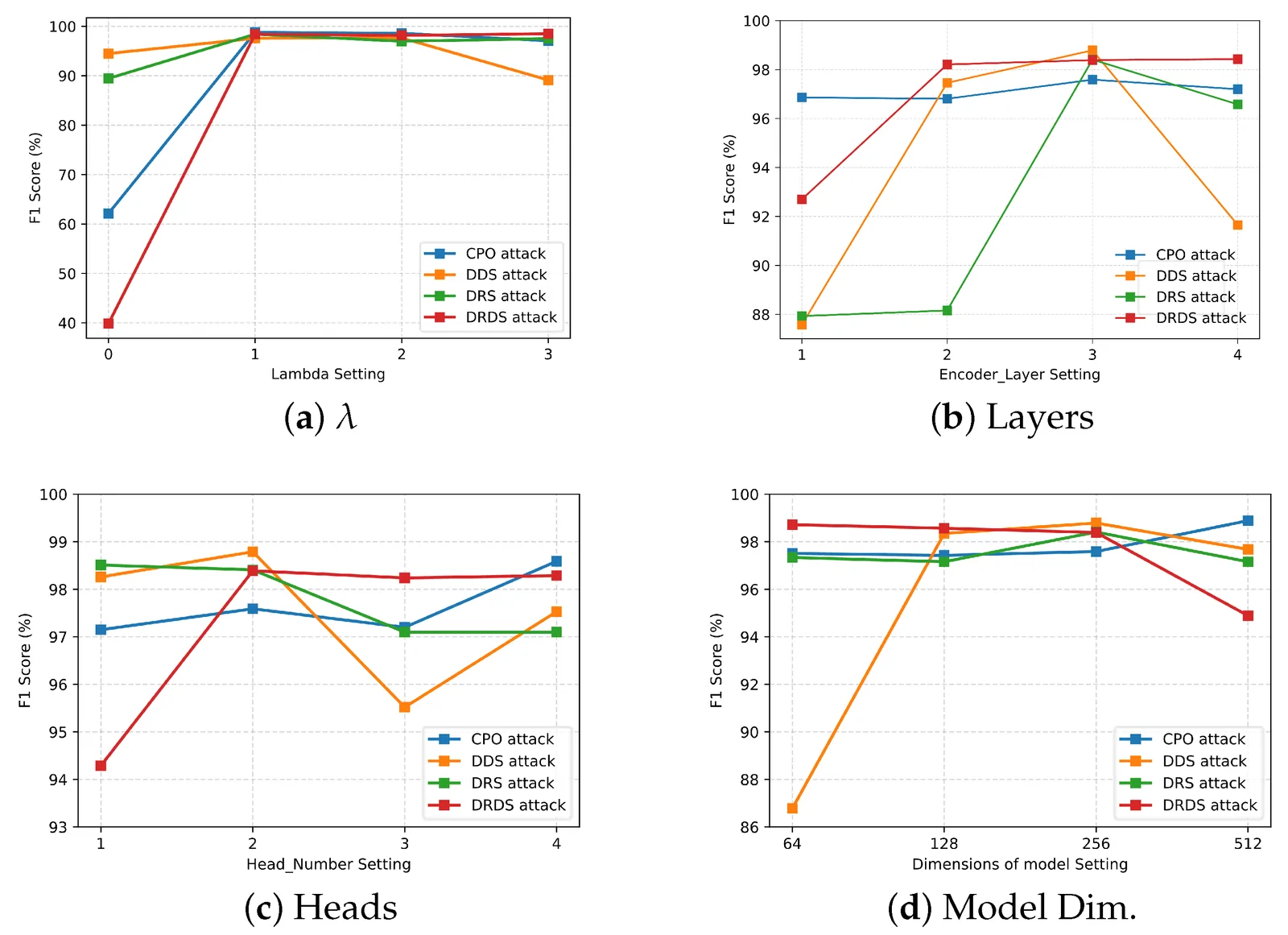
Parameter sensitivity studies of the main hyperparameters in SMRD.

The proposed SMRD model contains several hyperparameters that have a notable influence on its performance. To evaluate the robustness of SMRD under different parameter settings, we conducted a series of parameter sensitivity experiments. The results are shown in Figure 7.

Figure 7a shows that removing the Siamese term (λ=0) substantially reduces the F1-score, whereas λ=1 or 2 yields consistently high performance. Figure 7b indicates that three to four encoder layers provide the best accuracy–cost trade-off. Figure 7c,d show limited variation across the tested numbers of attention heads and model dimensions; we therefore use two heads and a dimension of 256.

#### 4.6.2. Visual Analysis

**Figure 8:**
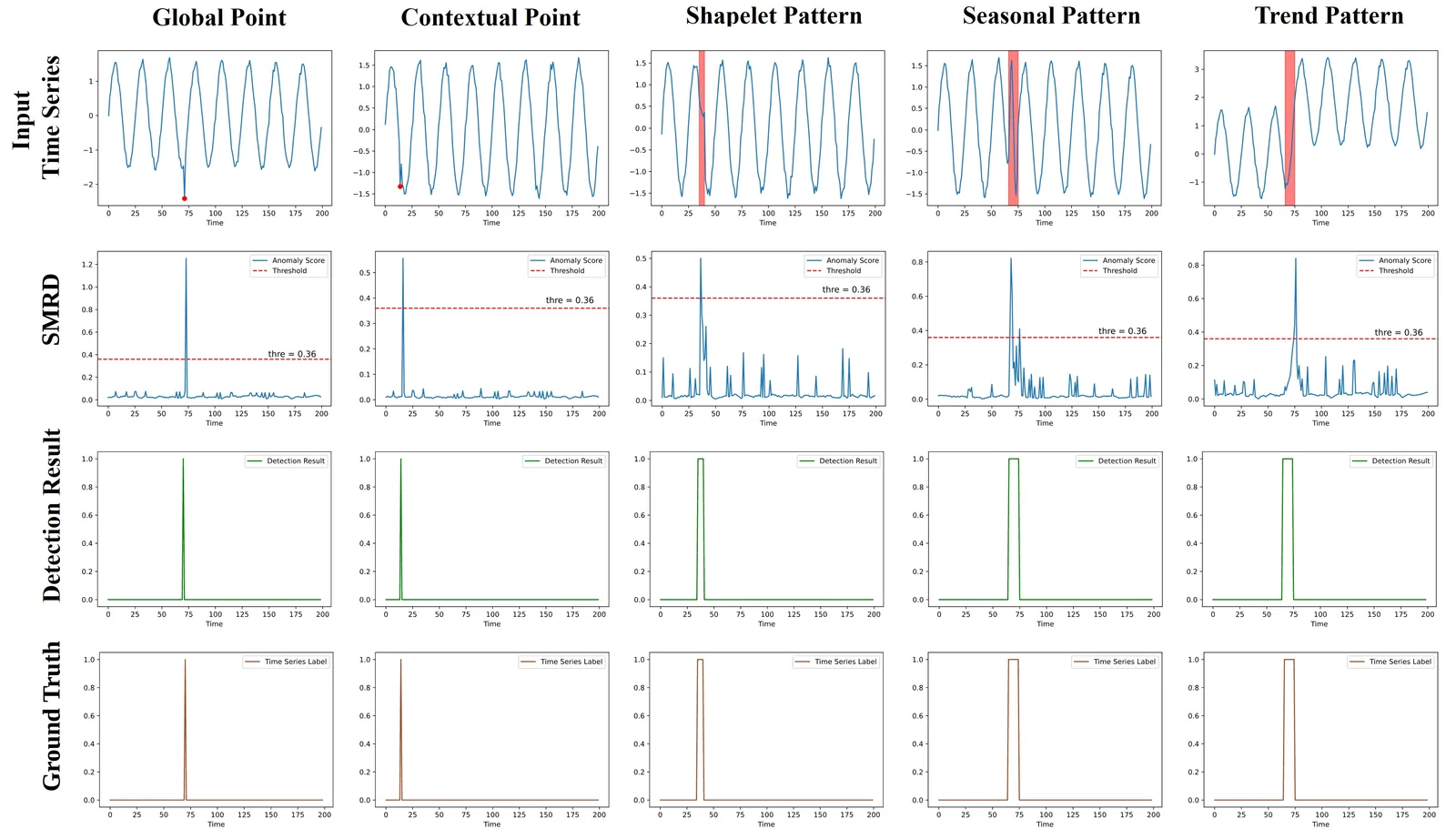
SMRD’s visualization process for different local anomalies. In the first row, the red shaded areas and red dots mark the injected pattern anomalies and point anomalies, respectively.

A key feature of SMRD is its ability to capture local dynamic information. To illustrate this capability, Figure 8 presents a visualization of the detection process. Following the data generation method proposed in [51], we constructed univariate time series containing different types of local anomalies, including point anomalies (global and contextual) and pattern anomalies (shapelet, seasonal, and trend anomalies). As shown in the figure, SMRD can effectively identify all these anomaly types, demonstrating its robustness in detecting diverse local abnormal patterns.

**Figure 9:**
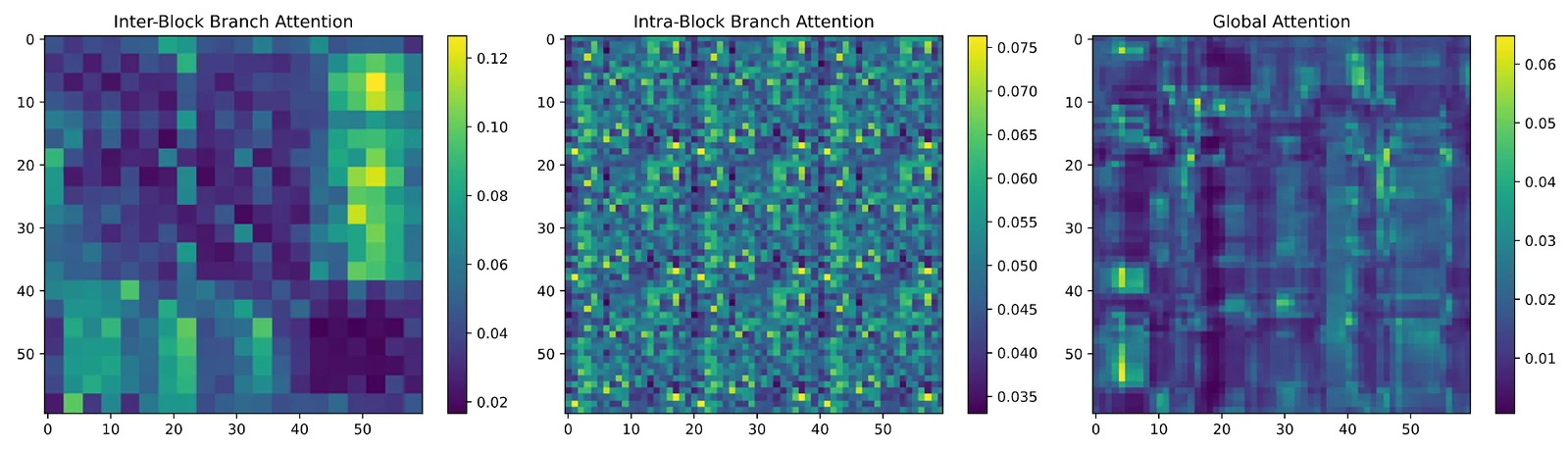
Visualization of global and local attention matrix.

In addition, we visualized the attention matrices corresponding to the global and local views, as shown in Figure 9. The global attention matrix primarily focuses on the overall temporal trends and long-range dependencies within the data. In contrast, the local attention matrix concentrates on regions containing local anomalies and short-term variations. This complementary attention mechanism enables the model to effectively exploit information from multiple views. As a result, SMRD can jointly capture global structural patterns and local abnormal behaviors, thereby improving the accuracy and robustness of attack detection.

### 4.7. Analysis in Practical Scenarios

#### 4.7.1. Real-Time Performance Analysis

In vehicular security and autonomous driving applications, computational complexity and inference latency are critical factors for real-world deployment on resource-constrained On-Board Units and Electronic Control Units. To assess the practical feasibility of SMRD, we compare it with four time-series anomaly detection baselines: TranAD, AnomalyBert, AnomalyTrans, and MAAT. The evaluation considers detection performance (F1-score), computational cost (FLOPs), model size (parameters), and inference latency. For a consistent comparison of detection performance, the reported F1-score is the macro-average over the four attack scenarios CPO, DDS, DRS, and DRDS.

As summarized in Table 8, SMRD achieves the highest average F1-score of 98.28%, outperforming the second-best method, MAAT, by 2.00 percentage points. SMRD requires 1.03 GFLOPs and 2.05M learnable parameters per forward pass. Although TranAD has lower computational cost and fewer parameters, SMRD remains more lightweight than AnomalyBert, AnomalyTrans, and MAAT while providing substantially better detection performance. SMRD also achieves the lowest measured inference latency among the compared methods. Latency is measured on an NVIDIA GeForce RTX 3080 GPU using a single window of length 60 with a batch size of 1. For SMRD, the average forward time is 7.04 ms (95th percentile: 7.32 ms); including adaptive normalization and the computation of the reconstruction and Siamese discrepancies, scoring one window takes 8.14 ms on average and 8.55 ms at the 95th percentile, which is the value reported in Table 8. This latency is below the 50 ms end-to-end target adopted in this study, leaving time for subsequent response and control operations. Overall, these results demonstrate that SMRD provides a favorable balance between detection accuracy, computational overhead, model size, and inference efficiency.

#### 4.7.2. Practical Deployment Feasibility in Vehicular Environments

Table 9 relates the measured computational cost and latency of SMRD to representative deployment constraints. The model requires 1.03 GFLOPs per forward pass and achieved a 95th-percentile scoring latency of 8.55 ms on a desktop GPU, reflecting the low computational cost of SMRD. These results support its computational feasibility for resource-constrained vehicular platforms.

## 5. Conclusions

We presented SMRD, a multi-view framework that combines global reconstruction discrepancy, local intra-block and inter-block Siamese discrepancy, and adaptive normalization for attack detection in IoV time series. Across four F2MD attack scenarios and three public benchmarks, SMRD achieved leading or competitive detection performance, while ablation results confirmed the complementary contributions of its global and local views. These findings support multi-view discrepancy learning for localized vehicular attacks. In future work, we will extend SMRD with longer-horizon temporal modeling and with predictive forecasting and generative reconstruction to improve early-stage threat detection, and we will further evaluate it on additional attack families, real V2X data, and on-board platforms.

## Figures and Tables

**Table 1:** F2MD simulation parameters.

Simulation Parameters	Value
Road network topology	LuSTNanoScenario
Simulation time	5 min
Pseudonym Change Protocol	Car2Car
Vehicle motion model	Random
Communication protocol	IEEE 802.11p/1609
Attack vehicle density	20%, 35%
Attack types	CPO, DDS, DRS, DRDS
Beacon transmission frequency	1 Hz
Obstacle decay model	Simple

**Table 2:** Description of attack types in the dataset.

Attack Types	Description	Effect on Time Series
CPO	The attacker vehicle broadcasts its real position with a constant offset limited to a maximum value ($\Delta (0\to P_{\max})$)	Persistent offset of position values
DDS	The attacker vehicle performs a disruptive DoS attack while changing its pseudonym for each vehicle-to-everything (V2X) message it sends	Abnormal message frequency and frequent identity changes
DRS	The attacker vehicle performs a data replay attack while simultaneously changing its pseudonym and target vehicle	Temporal replay of previously observed messages
DRDS	The attacker vehicle performs a DoS attack while simultaneously randomizing all V2X message data	Random message values and abnormal message frequency

**Table 3:** Experimental settings for all datasets. AR (anomaly ratio) represents the abnormal proportion of the test set.

	F2MD Attack Scenarios				Public Benchmarks		
Setting	CPO	DDS	DRS	DRDS	SPMD	MSL	SMAP
Block_Size	[3,5]	[3,5]	[3,5]	[3,5]	[3,5]	[3,5]	[3,5]
Win_Size	60	60	60	60	60	60	60
Threshold δ(%)	1	1	1	2	1	1	1
Training	14,000	14,000	14,000	14,000	64,653	58,317	135,183
Test (Labeled)	5101	3944	4387	4996	8922	73,729	427,617
AR (%)	21.06	35.37	21.13	35.62	10.52	10.72	13.13

**Table 4:** Overall comparative performance on multivariate time-series datasets (in %). Bold values indicate best results; underlined values indicate second-best.

Dataset	CPO			DDS			DRS			DRDS		
Metric	P	R	F1	P	R	F1	P	R	F1	P	R	F1
iForest	32.98	66.25	44.04	39.29	66.41	49.37	31.32	60.22	41.21	47.47	46.74	47.10
OCSVM	33.56	76.67	46.69	40.47	52.37	45.66	45.8	54.85	49.92	48.27	69.36	56.92
DAGMM	79.78	81.04	80.41	72.49	87.56	79.32	76.33	81.44	78.80	77.81	74.16	75.94
OmniAnomaly	84.36	78.88	81.53	84.73	76.53	80.42	83.21	80.62	81.89	80.99	81.48	81.23
MAD-GAN	84.74	68.45	75.73	83.57	81.35	82.45	81.25	76.49	78.80	86.48	82.54	84.46
CAE-M	88.16	85.14	86.62	86.34	82.96	84.62	83.15	76.45	79.66	89.47	83.18	86.21
TranAD	90.87	83.48	87.02	92.54	92.48	92.51	92.16	80.44	85.90	88.12	80.15	83.95
DTAAD	95.75	99.25	97.47	97.45	97.19	97.32	94.45	84.15	89.00	85.35	72.73	78.54
AnomalyBert	**98.94**	90.45	94.50	**99.28**	86.30	92.34	91.22	92.54	91.88	96.52	86.42	91.19
AnomalyTrans	96.99	99.89	98.42	96.59	90.77	93.59	96.58	**99.69**	98.11	96.58	86.32	91.16
UltraADV	95.42	94.11	94.76	94.56	95.82	95.19	95.88	93.42	94.63	87.41	97.13	92.01
ET-DRF-GA	97.88	96.45	97.16	96.72	96.15	96.43	**97.42**	95.38	96.39	96.40	90.11	93.15
Ours	97.68	**99.92**	**98.79**	97.87	**97.31**	**97.59**	97.35	99.48	**98.40**	**97.36**	**99.37**	**98.35**

**Table 5:** Evaluation under advanced range-based and volume-based metrics across four attack scenarios (%). A higher value indicates superior performance. Bold indicates the best result; underline indicates the second-best.

Dataset	Model	Aff-P	Aff-R	R_A_R	R_A_P	V_ROC	V_PR
CPO	AnomalyBert	**88.53**	**98.91**	76.13	72.00	78.24	74.07
	DTAAD	56.15	84.49	89.46	86.59	91.56	91.48
	AnomalyTrans	57.19	86.88	91.93	93.69	92.62	94.22
	Ours	79.53	96.96	**92.28**	**94.66**	**93.02**	**95.19**
DDS	AnomalyBert	**99.87**	69.2	67.69	70.15	68.94	71.36
	DTAAD	92.16	72.16	84.26	90.56	82.46	91.24
	AnomalyTrans	66.45	71.47	82.26	90.18	81.12	89.35
	Ours	93.91	**73.64**	**84.52**	**91.66**	**85.91**	**92.39**
DRS	AnomalyBert	**88.21**	**97.73**	78.94	77.31	79.67	78.00
	DTAAD	62.16	79.16	78.16	79.48	76.15	77.16
	AnomalyTrans	64.46	87.31	80.70	87.52	80.98	**87.78**
	Ours	84.49	93.00	**83.07**	**87.91**	**83.44**	87.25
DRDS	AnomalyBert	54.76	72.54	85.57	90.37	84.43	88.86
	DTAAD	49.49	68.41	80.41	85.16	78.15	79.83
	AnomalyTrans	55.16	72.62	84.29	89.86	84.48	89.93
	Ours	**61.50**	**92.09**	**93.26**	**96.37**	**92.92**	**96.13**

**Table 6:** Comparative evaluation on public real-world datasets (SPMD, MSL, and SMAP) in %. Bold indicates the best result; underline indicates the second-best.

Dataset	SPMD			MSL			SMAP		
Metric	P	R	F1	P	R	F1	P	R	F1
OmniAnomaly	90.94	84.2	87.44	89.02	86.37	87.67	92.49	81.99	86.92
InterFusion	85.40	90.65	87.95	81.28	92.7	86.62	89.77	88.52	89.14
THOC	90.39	90.10	90.24	88.45	90.97	89.69	92.06	89.34	90.68
BeatGAN	90.91	80.66	85.48	89.75	85.42	87.53	92.38	55.85	69.61
Adformer	92.62	93.23	92.92	92.00	88.64	90.29	95.67	97.64	96.64
TranAD	85.20	**95.13**	89.89	90.37	95.69	92.95	80.43	**99.99**	89.15
AnomalyTrans	92.67	93.78	93.22	91.92	96.03	93.93	93.59	99.41	96.41
MUTANT	93.24	94.98	94.10	**92.84**	96.44	94.61	97.88	97.19	97.53
CARLA	88.13	92.41	90.22	85.45	96.50	90.64	73.42	99.35	84.44
MAAT	90.87	94.23	92.52	92.06	**98.33**	**95.09**	94.75	99.33	96.99
Ours	**93.49**	95.03	**94.25**	92.82	96.62	94.68	**98.27**	99.61	**98.94**

**Table 7:** Comparison among SMRD variants. Unilateral denotes a two-stage adaptive normalization strategy, while Bilateral denotes a one-stage strategy. Standard attn. replaces the Siamese branch with a standard attention branch. Bold indicates the best result; underline indicates the second-best.

Architecture	Ablation Settings			Attack Types				Avg F1 (%)
	Reconstruction Discrepancy	Siamese Discrepancy	Normalization Strategy	CPO	DDS	DRS	DRDS	
Variants	✘	✔	✘	94.61	91.27	96.58	98.14	95.15
	✔	✘	✘	60.37	93.84	78.53	36.41	67.29
	✔	✘	Bilateral	62.13	94.49	89.45	39.85	71.48
	✔	✔	Unilateral	93.09	96.57	64.11	98.07	87.96
	✔	✔	✘	82.17	95.83	94.69	98.21	92.73
	✔	Standard attn.	Bilateral	93.83	91.22	92.30	93.15	92.63
Ours	✔	✔	Bilateral	**98.79**	**97.59**	**98.40**	**98.35**	**98.28**

✔ indicates that the component is included; ✘ indicates that the component is not included.

**Table 8:** Comparison of detection performance and computational efficiency. FLOPs and Params denote the floating-point operations per forward pass and the number of learnable parameters, respectively. F1 is the macro-average over the CPO, DDS, DRS, and DRDS attack scenarios. Bold indicates the best result; underline indicates the second-best. Lower values are better for FLOPs, Params, and Latency, whereas higher values are better for F1.

Model	FLOPs (G)	Params (M)	F1 (%)	Latency (ms)
TranAD	**0.57**	**1.51**	87.35	11.59
AnomalyBert	1.11	10.05	92.48	8.71
AnomalyTrans	1.05	3.88	95.32	9.68
MAAT	1.28	7.02	96.28	10.02
Ours	1.03	2.05	**98.28**	**8.55**

**Table 9:** Systemic feasibility analysis of deploying SMRD in real-world vehicular environments.

Challenge Dimension	Real-World Constraints	SMRD Deployment Capabilities
Computational Resources	Limited in-vehicle computing power (2× NVIDIA Orin-X, 508 TOPS)	Low computational demand (about 1.03 GFLOPs per forward pass), compatible with TensorRT acceleration
Real-time Performance	End-to-end latency must remain below 50 ms	95th-percentile latency of 8.55 ms on a desktop GPU
Operational Stability	Frequent and high-density attack scenarios	F1-score above 80% for all attack types with up to 60% attacks in the test data (Section 4.4.3)
Privacy Compliance	Regulatory constraints, such as EU GDPR, on data transmission	Performs on-device anomaly detection to reduce the risk of sensitive information leakage

## Data Availability

The data supporting the findings of this study are available from the corresponding author upon reasonable request. The study also uses publicly available benchmark datasets identified in the manuscript.

## References

[1] Tan J., Huang L., Xia Z., Gu K., Hao W., Long K., Zeng L. (2025). DATI-IDS: Domain adaptation and time-series imaging-based intrusion detection system for connected autonomous vehicles. IEEE Trans. Intell. Transp. Syst..

[2] Gao Z., Li X., Wei Z., Liu W., Guo G., Wen S. (2025). Observer-based secure predefined-time control of vehicular platoon systems under attacks in sensors and actuators. IEEE Trans. Intell. Transp. Syst..

[3] Haidar F., Makassikis M., Sall M., Bakhti H., Kaiser A., Lonc B. (2021). Experimentation and Assessment of Pseudonym Certificate Management and Misbehavior Detection in C-ITS. IEEE Open J. Intell. Transp. Syst..

[4] Park S., Kim D., Lee S. (2024). Enhancing V2X Security Through Combined Rule-Based and DL-Based Local Misbehavior Detection in Roadside Units. IEEE Open J. Intell. Transp. Syst..

[5] Vu L., Suo K., Islam M.R., Dhar N., Nguyen T.N., He S., Shi Y. Living on the Electric Vehicle and Cloud Era: A Study of Cyber Vulnerabilities, Potential Impacts, and Possible Strategies. Proceedings of the 2024 ACM Southeast Conference.

[6] Yahya M.A., Moya A.R., Ventura S. (2025). Deep learning for multivariate time series anomaly detection: An evaluation of reconstruction-based methods. Artif. Intell. Rev..

[7] Wei Y., Jang-Jaccard J., Xu W., Sabrina F., Camtepe S., Boulic M. (2023). LSTM-autoencoder-based anomaly detection for indoor air quality time-series data. IEEE Sens. J..

[8] Su L., Zuo X., Li R., Wang X., Zhao H., Huang B. (2025). A systematic review for transformer-based long-term series forecasting. Artif. Intell. Rev..

[9] Dai Y., Spence I., Rafferty K., Quinn B., Huang J., Wang H. (2025). TDSRL: Time series dual self-supervised representation learning for anomaly detection from different perspectives. IEEE Internet Things J..

[10] Nsour A., Ganesan S. (2026). Dynamic encryption based secure key management for Vehicle-to-Vehicle communication in autonomous driving. Sci. Rep..

[11] Nouretdinov I., Gammerman J., Fontana M., Rehal D. (2020). Multi-level conformal clustering: A distribution-free technique for clustering and anomaly detection. Neurocomputing.

[12] Hu W., Gao J., Li B., Wu O., Du J., Maybank S. (2018). Anomaly detection using local kernel density estimation and context-based regression. IEEE Trans. Knowl. Data Eng..

[13] Yang J., Chen Y., Rahardja S. (2023). Neighborhood representative for improving outlier detectors. Inf. Sci..

[14] Park D., Hoshi Y., Kemp C.C. (2018). A multimodal anomaly detector for robot-assisted feeding using an LSTM-based variational autoencoder. IEEE Robot. Autom. Lett..

[15] Su Y., Zhao Y., Niu C., Liu R., Sun W., Pei D. Robust anomaly detection for multivariate time series through stochastic recurrent neural network. Proceedings of the 25th ACM SIGKDD International Conference on Knowledge Discovery & Data Mining.

[16] Tuli S., Casale G., Jennings N.R. (2022). TranAD: Deep transformer networks for anomaly detection in multivariate time series data. Proc. VLDB Endow..

[17] Xu J., Wu H., Wang J., Long M. Anomaly Transformer: Time Series Anomaly Detection with Association Discrepancy. Proceedings of the International Conference on Learning Representations.

[18] Jeong Y., Yang E., Ryu J.H., Park I., Kang M. Anomalybert: Self-supervised transformer for time series anomaly detection using data degradation scheme. Proceedings of the International Conference on Learning Representations.

[19] Darban Z.Z., Webb G.I., Pan S., Aggarwal C.C., Salehi M. (2025). CARLA: Self-supervised contrastive representation learning for time series anomaly detection. Pattern Recognit..

[20] Sellam A.Z., Benaissa I., Taleb-Ahmed A., Patrono L., Distante C. (2025). Mamba Adaptive Anomaly Transformer with association discrepancy for time series. Eng. Appl. Artif. Intell..

[21] Pljonkin A., Rumyantsev K., Singh P.K. (2017). Synchronization in Quantum Key Distribution Systems. Cryptography.

[22] Pljonkin A., Petrov D., Sabantina L., Dakhkilgova K. (2021). Nonclassical Attack on a Quantum Key Distribution System. Entropy.

[23] Van Wyk F., Wang Y., Khojandi A., Masoud N. (2019). Real-time sensor anomaly detection and identification in automated vehicles. IEEE Trans. Intell. Transp. Syst..

[24] Alladi T., Gera B., Agrawal A., Chamola V., Yu F.R. (2021). DeepADV: A deep neural network framework for anomaly detection in VANETs. IEEE Trans. Veh. Technol..

[25] Khanmohammadi F., Azmi R. (2024). Time-Series Anomaly Detection in Automated Vehicles Using D-CNN-LSTM Autoencoder. IEEE Trans. Intell. Transp. Syst..

[26] Huang W., Xu H., Gong Y., Liu Z., Li F., Lin Z., Hu B.J. (2025). UltraADV: An Unsupervised Deep Learning Lightweight Framework for Anomaly Detection in V2X. IEEE Internet Things J..

[27] Eziama E.U., Kasm O.A., Nasir M. (2026). Enhanced Ensemble Temporal Convolutional Network With Genetic Algorithm Optimization for Anomaly Detection in CAVs. IEEE Trans. Intell. Transp. Syst..

[28] Li Z., Wu Y., Guang M., Jiang C. (2026). MPGTrans: Semi-Supervised Misbehavior Detection with Multi-Path Graph Transformer for Internet of Vehicles. IEEE Trans. Mob. Comput..

[29] Cai X., Huang D., Zhang G.Y., Wang C.D. (2023). Seeking commonness and inconsistencies: A jointly smoothed approach to multi-view subspace clustering. Inf. Fusion.

[30] Qin S., Chen L., Luo Y., Tao G. (2023). Multi-View Graph Contrastive Learning for Multivariate Time Series Anomaly Detection in IoT. IEEE Internet Things J..

[31] Guo M.H., Lu C.Z., Liu Z.N., Cheng M.M., Hu S.M. (2023). Visual attention network. Comput. Vis. Media.

[32] Bai Y., Wang X., Ang M.H., Rus D. (2022). BIMS-PU: Bi-Directional and Multi-Scale Point Cloud Upsampling. IEEE Robot. Autom. Lett..

[33] Chen X., He K. Exploring simple siamese representation learning. Proceedings of the IEEE/CVF Conference on Computer Vision and Pattern Recognition.

[34] Liu Y., Wu H., Wang J., Long M. (2022). Non-stationary transformers: Exploring the stationarity in time series forecasting. Adv. Neural Inf. Process. Syst..

[35] Kamel J., Wolf M., Van Der Hei R.W., Kaiser A., Urien P., Kargl F. Veremi extension: A dataset for comparable evaluation of misbehavior detection in vanets. Proceedings of the ICC 2020—2020 IEEE International Conference on Communications (ICC).

[36] Kamel J., Ansari M.R., Petit J., Kaiser A., Jemaa I.B., Urien P. (2020). Simulation framework for misbehavior detection in vehicular networks. IEEE Trans. Veh. Technol..

[37] Zhao X., Wu Y., Lee D.L., Cui W. (2018). iforest: Interpreting random forests via visual analytics. IEEE Trans. Vis. Comput. Graph..

[38] Hameed M.A., Khalaf Z.A. (2024). An Approach for Aircraft Detection using VGG19 and OCSVM. Int. J. Comput. Digit. Syst..

[39] Li D., Chen D., Jin B., Shi L., Goh J., Ng S.K. MAD-GAN: Multivariate anomaly detection for time series data with generative adversarial networks. Proceedings of the International Conference on Artificial Neural Networks.

[40] Zhang Y., Chen Y., Wang J., Pan Z. (2021). Unsupervised deep anomaly detection for multi-sensor time-series signals. IEEE Trans. Knowl. Data Eng..

[41] Yu L.R., Lu Q.H., Xue Y. (2024). DTAAD: Dual TCN-attention networks for anomaly detection in multivariate time series data. Knowl.-Based Syst..

[42] Zong B., Song Q., Min M.R., Cheng W., Lumezanu C., Cho D., Chen H. Deep autoencoding gaussian mixture model for unsupervised anomaly detection. Proceedings of the International Conference on Learning Representations.

[43] Zhou M., Han L., Wang J. (2024). Physical invariant subspace based unsupervised anomaly detection for internet of vehicles. IEEE Trans. Intell. Veh..

[44] Hundman K., Constantinou V., Laporte C., Colwell I., Soderstrom T. Detecting spacecraft anomalies using LSTMs and nonparametric dynamic thresholding. Proceedings of the 24th ACM SIGKDD International Conference on Knowledge Discovery & Data Mining.

[45] Wen J., Jiang D., Tu G., Liu C., Cambria E. (2023). Dynamic interactive multiview memory network for emotion recognition in conversation. Inf. Fusion.

[46] Zeng F., Chen M., Qian C., Wang Y., Zhou Y., Tang W. (2023). Multivariate time series anomaly detection with adversarial transformer architecture in the Internet of Things. Future Gener. Comput. Syst..

[47] Shi Y., Wang B., Yu Y., Tang X., Huang C., Dong J. (2023). Robust anomaly detection for multivariate time series through temporal GCNs and attention-based VAE. Knowl.-Based Syst..

[48] Shen L., Li Z., Kwok J. (2020). Timeseries anomaly detection using temporal hierarchical one-class network. Adv. Neural Inf. Process. Syst..

[49] Li Z., Zhao Y., Han J., Su Y., Jiao R., Wen X., Pei D. Multivariate time series anomaly detection and interpretation using hierarchical inter-metric and temporal embedding. Proceedings of the 27th ACM SIGKDD Conference on Knowledge Discovery & Data Mining.

[50] Zhou B., Liu S., Hooi B., Cheng X., Ye J. Beatgan: Anomalous rhythm detection using adversarially generated time series. Proceedings of the IJCAI.

[51] Lai K.H., Zha D., Xu J., Zhao Y., Wang G., Hu X. Revisiting time series outlier detection: Definitions and benchmarks. Proceedings of the Thirty-Fifth Conference on Neural Information Processing Systems.

